# Toxic Elements in Beans from Zhejiang, Southeast China: Distribution and Probabilistic Health Risk Assessment

**DOI:** 10.3390/foods12173300

**Published:** 2023-09-02

**Authors:** Sha Yu, Xiao-Dong Pan, Jian-Long Han

**Affiliations:** Zhejiang Provincial Center for Disease Control and Prevention, Hangzhou 310051, China

**Keywords:** heavy metals, beans, Monte Carlo, health risk assessment, target hazard quotients

## Abstract

This study described the distribution of As, Cd, Cr, Hg, and Pb in 692 bean samples from Zhejiang province, southeast China, and estimated the health risk using Monte Carlo simulation. The average levels of As, Cd, Cr, Hg, and Pb were 0.0349, 0.0379, 0.246, 0.0019, and 0.0246 mg kg^−1^. Correlation analyses showed very strong positive correlations for Cd-Pb in kidney beans and mung beans, Cd-As in black beans, and Pb-As in red beans. The target hazard quotients (THQs) were adopted for non-carcinogenic risk assessment, and THQs at the 50th percentile were all less than 1, indicating that there are no deleterious effects from rice exposure to these elements. When evaluating THQ for multiple elements, the certainty with a hazard index (HI) greater than 1 for children was 12.64%, for teens 11.54%, and for adults 1.01%. The sensitivity analysis reveals that the concentration of Cd in beans and ED (exposure duration) are the main principal factors that contributed to the total risk. The mean carcinogenic risks for children, teens, and adults were all less than 1 × 10^−4^, indicating no potential carcinogenic risk. Despite that, the routine monitoring of these elements, especially for Cd should be continued.

## 1. Introduction

Beans, which include various legume species such as soybeans, mung beans, and black beans, are widely cultivated and consumed in Asian countries, especially in China. They serve as a valuable source of dietary protein and essential nutrients for the local population. However, the extensive cultivation of beans in areas affected by industrial activities raises concerns about the potential accumulation of heavy metals in these crops.

Potentially toxic elements, such as lead (Pb), cadmium (Cd), arsenic (As), and mercury (Hg), are among the most common contaminants found in agricultural products. In China, a recent study revealed the potential for Cd pollution in vegetables, especially bulb vegetables [1]. In Bangladesh, leafy vegetables cultivated on tannery effluent-contaminated soil had higher metal contents than other agricultural soils, particularly in the case of Cr and Cd [2]. Similarly, in India, it was reported that there were high levels of Cd and Pb in rice grains grown in areas near industrial sites [3]. In Germany, beans, carrots, and lettuce grown in the gardens of a mine dump area showed high concentrations of arsenic (As), copper (Cu), manganese (Mn), mercury (Hg), lead (Pb), and zinc (Zn) [4].

The accumulation of toxic elements in agricultural products involves several factors, including soil composition, irrigation water, and fertilizers. The accumulation is influenced by physiological and biochemical processes, such as root anatomy, nutrient competition, and defense mechanisms [5,6]. Furthermore, genetic factors and environmental conditions play a role in determining the uptake and accumulation of toxic elements. For instance, Cadmium accumulation in plants can increase with decreasing soil pH [7].

Some elements, such as Pb, Cd, As, and Hg, have detrimental health effects on the human body [8]. Lead exposure can cause cognitive impairment, developmental delays in children, and nervous system disorders. Cadmium exposure is associated with kidney damage, osteoporosis, and lung cancer. Arsenic exposure can lead to various health problems, including skin lesions, cardiovascular diseases, and an increased risk of cancer. Mercury exposure can affect the nervous system, leading to neurological symptoms, developmental issues in children, and impairments in cognitive function. Therefore, the analysis of heavy metal distribution in crops receives increasing concern from both scientists and consumers.

Zhejiang Province located in the southeast of China, has an area of 105,500 square kilometers and has about 65 million residents. The region has experienced a significant surge in urbanization and industrialization, resulting in a substantial GDP of approximately 750 billion CNY by 2022. However, this rapid development raises concerns regarding the potential contamination of agricultural products with heavy metals. Our previous studies have revealed the possible risks of heavy metal contamination in vegetables, rice, meat, and marine products [1,9,10,11,12,13,14]. However, to our knowledge, in the past two years, there have been no reports on heavy metals or probabilistic risk assessments for beans from Zhejiang province, southeast China.

The main objective of this study was to investigate the distribution of As, Cd, Cr, Hg, and Pb in different beans and assess the potential health risks associated with daily consumption. This will involve evaluating the levels of heavy metals in bean samples collected from various regions, characterizing the correlation of these elements, comparing the sampling regions, and assessing the potential health risks using Monte Carlo simulation (MCS).

## 2. Materials and Methods

### 2.1. Sampling

Bean samples (*n* = 692) were purchased from local commercial markets in eleven monitoring areas (Hangzhou, Huzhou, Jiaxing, Jinhua, Lishui, Ningbo, Quzhou, Shaoxing, Taizhou, Wenzhou, and Zhoushan) in Zhejiang Province, Southeastern China. Black bean (*Phaseolus vulgaris* L.), broad bean (*Vicia faba* L.), mung bean (*Vigna radiata* L.), soybean (*Glycine max*), red bean (*Phaseolus angularis*), kidney bean (*Phaseolus vulgaris*), and pea (*Pisum sativum* L.) were collected. The beans were all dried samples with a water content of not more than 15%. Following collection, the samples were ground into powders and stored at −20 °C until they underwent laboratory testing.

### 2.2. Chemicals

All solutions were prepared with analytical reagent-grade chemicals and deionized Milli-Q water (Millipore, Bedford, MA, USA). And 65% nitric acid (Suprapur grade, Merck, Darmstadt, Germany) was used for sample dissolution. Standard solutions were prepared by appropriate dilution of 1000 mg L^−1^ multi-element CertiPur grade standards (Merck, Darmstadt, Germany).

### 2.3. Analysis and Quality Control

The analysis of heavy metals was carried out by laboratories affiliated with the Centers for Disease Control and Prevention (CDC) in cities in Zhejiang province, including Hangzhou, Huzhou, Jiaxing, Jinhua, Lishui, Ningbo, Quzhou, Shaoxing, Taizhou, Wenzhou, and Zhoushan. The analytical method employed in this study was based on previous reports [1,13]. In summary, ground samples were digested using closed microwave-assisted digestion with nitric acid (HNO_3_). Following digestion, the residue was dried at 150 °C until nearly dry and then re-diluted with de-ionized water to a final volume of 20 mL for instrumental analysis. Inductively coupled plasma-mass spectrometry (ICP-MS) was utilized to test for arsenic (As), cadmium (Cd), chromium (Cr), mercury (Hg), and lead (Pb) in all samples.

To ensure the quality control of the laboratory analysis, a certified reference material (CRM) of soybean flour (GBW10013) was used. The tested values of all elements in the CRMs were within a 10% deviation from the certified values (Table 1). The limit of detection (LOD) is the concentration at which the signal level of the substance reaches at least three times the signal noise of the baseline. The limits of quantification (LOQ) can be determined by a signal-to-noise ratio of 10:1 or approximated by multiplying the LOD by 3.3.

### 2.4. Exposure Assessment

The test data were prepared in accordance with the Assessment Method for Low Content Pollutants in Food outlined by the World Health Organization (WHO) in 1995 (WHO, 1995) [15]. Specifically, when the proportion of undetected samples is less than or equal to 60% of the total number of samples, the undetected values are expressed as half of the LODs.

Exposure assessment plays a crucial role in providing essential data for health risk assessment. In this study, the estimated daily intake (EDI) for dietary exposure was calculated using Equation (1) [13].

(1) $$\mathrm{EDI}=\frac{C_{\mathrm{metal}}\times \mathrm{EF}\times \mathrm{ED}\times \mathrm{IR}\times f}{\mathrm{BW}\times \mathrm{AT}\times 1000}$$

where EDI (mg/kg/day) is the daily metal intake by consumption of beans and *C*_metal_ is concentration of elements in beans (mg/kg). IR is the food ingestion per day for the bean (g/day). IRs for children, teens, and adults were 32.9 g, 58.87 g, and 42.75 g, respectively [16]. BW is the reference bodyweight (kg). For children, teens, and adults, the BW was 16.68 ± 1.48 kg, 46.25 ± 1.18 kg, and 57.03 ± 1.10 kg, respectively [17]. The AT, average exposure time (day) of children, teens, and adults were 2190, 5475, and 18,250 days, respectively [18]. EF is the exposure frequency to the trace element (day/year), and ED is the exposure duration (year) [18].

### 2.5. Non-Carcinogenic Risk

To calculate the non-carcinogenic health risk of metal intake by rice for local residents, the target hazard quotient (THQ) was adopted [19]. THQ was calculated using Equation (2).

(2) $$\mathrm{THQ}=\frac{\mathrm{EDI}}{\mathrm{RfD}}$$

wherein RfD is the reference dose of elements. The RfDs of As, Cd, Cr, and Hg are 0.0003, 0.0001, 1.5, and 0.0001 mg/kg per day, as provided by the US EPA [20]. For Pb, a tolerable daily intake (TDI) equal to 0.0036 mg/kg per day, as proposed by FAO/WHO [21], was used. With respect to a RfD of 0.0003 mg/kg/day only for inorganic As, we transferred the concentration of total As to inorganic As. Approximately 72% of the total As in crops from China were the inorganic As species (As(III) + As(V)) [22].

The hazard index (HI) is defined as the sum of the individual THQs (Equation (3)). When the calculated value of HI is equal to or less than 1, there is no risk of a non-carcinogenic effect on health. And if the value is higher than 1, it would show non-carcinogenic impacts on human health [18].

(3) $$\mathrm{HI}=\sum_{i=1}^{n}\mathrm{THQ}$$

### 2.6. Carcinogenic Risk

When a person is exposed to a chemical or other agent that can damage DNA, we use carcinogenic risk (CR) to represent the probability of a person suffering cancer over a lifetime. The CR was calculated by the following Equation (4) [23].

CR = EDI × SF(4)

where EDI (mg/kg/day) is the estimated daily intake of the carcinogen element and SF (mg/kg/day) is the slope factor of the carcinogen element. In the present study, As was calculated for carcinogenic risk considering available toxicology information. The SF of inorganic As is 1.5/mg/kg/day. We took the value of 72% of the total As as the inorganic As.

### 2.7. Monte Carlo Simulation

Risk assessment is typically conducted using deterministic and stochastic approaches. Deterministic approaches rely on fixed input parameters and assume that the system being assessed will exhibit predictable behavior. Conversely, stochastic approaches take into account the inherent randomness of input parameters and utilize probability distributions to model uncertainty [24]. A widely employed technique for stochastic risk assessment is Monte Carlo simulation (MCS), which involves generating random values for input parameters and running simulations of the system multiple times to estimate the probability distribution of output variables [25].

In this study, we employed MCS by incorporating probability distributions of inputs and random numbers. The parameter distributions required for evaluating the probability functions in the risk assessment model for THQ (target hazard quotient) were derived from previous reports [16,26] and are provided in Table 2. The MCS was conducted for 5000, 10,000, and 50,000 iterations. However, it was observed that 10,000 iterations yielded reliable results. The simulation was carried out using Oracle Crystal Ball (version 11.1.34190, free trial version).

### 2.8. Statistical Analysis

Statistical analysis was conducted using SAS JMP 10.0 software (free trial version). To calculate the statistical differences in elemental content across different regions, we employed the Kruskal–Wallis non-parametric test method with a significance level (alpha) set at 0.05 for all statistical comparisons. Hierarchical cluster analysis was performed using Ward’s minimum variance method.

## 3. Results and Discussion

### 3.1. Method Validation for Elemental Analysis

In order to ensure the accuracy of laboratory analysis, the certified reference material (GBW10013) for rice was used. The values obtained for all elements in the certified reference material were within 10% of the certified values. To determine the method limit of detection (LOD), the concentration value corresponding to three times the standard deviation of 10 blank measurements was multiplied by the dilution volume (10 mL) and divided by the sample weight (0.5 g) (Table 1).

The standard solution linearity range for As, Cd, Cr, and Pb was set at 0 to 300 μg/L, while for Hg it was set at 0 to 3 μg/L. The linear correlation coefficients (*r*) for these analytes exceeded 0.995. All samples were digested and analyzed using instrumental methods coupled with certified reference materials (*n* = 20). The recovery of the analytes was calculated by dividing the obtained concentration by the certified concentration. The relative standard deviation (RSD) among the analyzed values was also calculated. The mean recoveries for As, Cd, Cr, and Pb ranged from 85% to 100%. The mean recovery for Hg was 80%. The RSDs for these analytes were all below 15%.

### 3.2. Levels of Potential Toxic Elements in Bean Samples

The average concentrations of elements in 692 bean samples were tested for Cd (0.0379 ± 0.014 mg/kg), Cr (0.246 ± 0.123 mg/kg), Hg (0.0019 ± 0.0017 mg/kg), Pb (0.0246 ± 0.011 mg/kg), and As (0.0349 ± 0.019 mg/kg). Table 3 presents the concentrations of Cd, Cr, Hg, Pb, and As in different beans collected from 11 monitoring areas in Zhejiang Province. Among them, the highest mean concentrations were found for Cd (0.0498 mg/kg) in soybean, Cr (0.322 mg/kg) in black bean, Hg (0.0027 mg/kg) in pea, Pb (0.0498 mg/kg) in kidney bean, and As (0.0601 mg/kg) in black bean. For the health of consumers, the maximum limits (ML) of these elements in beans have been established. The Joint FAO/WHO Expert Committee established the ML of elements in beans with Cd and Pb at 0.2 mg/kg [27]. In China, the ML of elements in beans is 0.1 mg/kg for Cd, 0.2 mg/kg for Pb, and 1.0 mg/kg for Cr [28]. As shown in Table 3, all types of beans except pea and kidney beans had a level of Cd exceeding the ML. For Cr, 3.6% of black bean samples, 2.2% of broad beans, 2.9% of soybeans, and 8.3% of kidney beans had concentrations exceeding the ML. Actually, Cr is an essential trace element in the human body that plays various roles. It contributes to glucose metabolism by enhancing insulin action, making it beneficial for individuals with diabetes or insulin resistance. However, exposure to high levels of toxic forms of Cr, such as hexavalent Cr found in industrial settings, can have harmful effects. Prolonged exposure to hexavalent Cr can lead to respiratory problems, skin irritation, and an increased risk of lung cancer. Moreover, different forms of mercury and arsenic have distinct toxic characteristics. Elemental mercury primarily causes neurological damage, while inorganic mercury compounds can lead to kidney damage and respiratory issues. Organic mercury compounds, such as methyl mercury, are particularly harmful to the central nervous system, especially during brain development. In contrast, inorganic forms of arsenic are associated with skin lesions, increased cancer risk, cardiovascular and respiratory problems, as well as neurotoxic effects. Organic forms of arsenic are generally considered to be less toxic.

Recent studies also revealed some heavy metals in different types of beans. Smith and Mohammed [29] found that Cr concentrations in the kidney beans and chickpeas from Trinidad and Tobago were within the ranges of 0.72–3.56 mg/kg and 1.73–2.44 mg/kg, respectively. Legumes in Iraq showed concentrations ranging from 0.039 to 0.369  mg/kg for Pb, 0.005–0.052  mg/kg for Cr, and 0.001–0.191  mg/kg for As. Hg and Cd were not detected (<0.001  mg/kg) in the legume samples [30]. Brown beans from Nigeria had concentrations of Cd and Pb ranging from 0.001 to 0.01 mg/kg and 0.001 to 1.52 mg/kg, respectively [31]. Islam et al. [32] tested the lentils consumed in Bangladesh for Cr, As, Cd, and Pb with mean concentrations of 0.68 mg/kg, 0.75 mg/kg, 0.03 mg/kg, and 0.31 mg/kg, respectively. Legume samples from Iran contained 0.035 mg/kg of Hg and 0.562 mg/kg of Pb [33].

**Figure 1:**
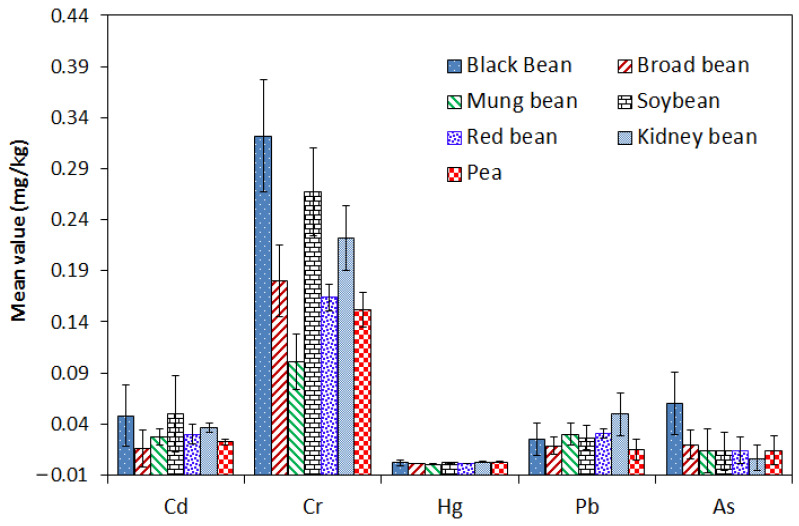
The mean concentration of As, Cd, Cr, Hg, and Pb in different beans.

In our study, we found that levels of Cr and As in black beans were significantly higher than in other types of beans (*p* < 0.05) (Figure 1). The kidney bean had the highest mean concentration of Pb. Other researchers also reported that different legumes had diverse concentrations of metals, including cadmium (Cd), lead (Pb), zinc (Zn), copper (Cu), arsenic (As), aluminum (Al), molybdenum (Mo), manganese (Mn), mercury (Hg), and chromium (Cr) [30]. They indicate that different species of beans have a diverse capacity for heavy metal accumulation. The uptake of heavy metals by plants depends on several factors, including soil properties such as pH, phosphate, and organic matter. Furthermore, high concentrations of heavy metals could be toxic to plants due to the increased generation of reactive oxygen species and oxidative stress [7].

### 3.3. The Correlation Analysis of Different Elements

Spearman correlation analyses were performed to reveal relationships among different elements in beans (Supplemental Tables). The degree of correlation was assessed using the correlation coefficient (*r*), which indicated the strength of the relationship. The strength of the correlation was categorized as weak (0.01–0.39), moderate (0.40–0.59), strong (0.60–0.79), or very strong (0.80–1). For all beans, a moderately positive correlation between Cd-Pb (*r* = 0.399, *p* < 0.01) and Cd-As (*r* = 0.524, *p* < 0.01) was observed. Additionally, we found a very strong positive correlation between Cd and Pb (*r* = 0.934, *p* < 0.01) in kidney beans. Strong positive correlation of Cd-As (*r* = 0.720, *p* < 0.01) in black beans, Pb-As (*r* = 0.701, *p* < 0.01) in red beans, Cd-Pb (*r* = 0.792, *p* < 0.01) in mung beans, and Cd-Hg (*r* = 0.746, *p* < 0.01) in kidney beans. Previous studies have also shown that Cd, Pb, and Zn are significantly correlated in the roots, stems, leaves, and fruits of asparagus beans [34].

There are multiple potential mechanisms that may elucidate the correlations observed among various elements in beans [7]. Firstly, positive correlations could arise from their concurrent existence in identical geological formations, resulting in their accumulation within the same samples. Then, common exposure pathways may lead to positive correlations between certain elements. Negative correlations among specific elements in bean samples might result from competitive adsorption, where multiple elements vie for the same adsorption sites. Biological uptake and transport processes could also contribute to the correlations witnessed between diverse elements in beans.

### 3.4. Spatial Distribution of Sampling Areas

**Figure 2:**
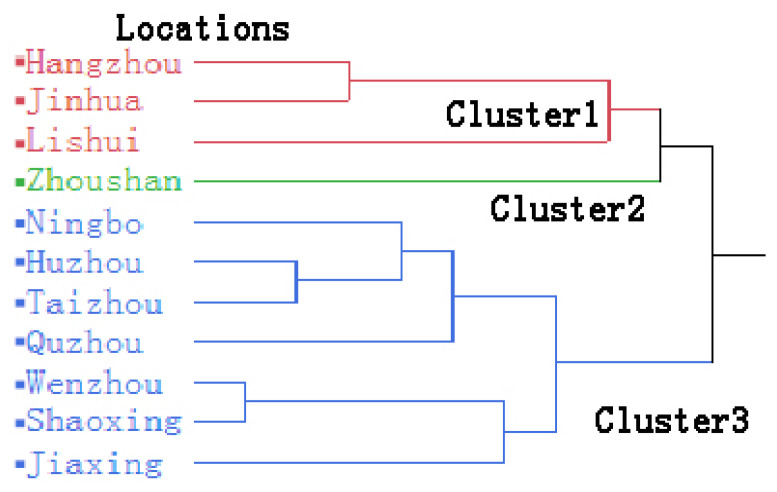
The hierarchical cluster analysis of areas based on metal concentrations in beans from Zhejiang, southeast China.

Based on the levels of As, Cd, Cr, Hg, and Pb in beans, a hierarchical cluster analysis was performed and presented in Figure 2. The eleven sampling areas were divided into three clusters. It showed that three areas of Hangzhou, Jinhua, and Lishui were classified into Cluster 1 (C1), Zhoushan was classified as Cluster 2 (C2), and the left areas were classified as Cluster 3 (C3). The location of Zhoushan was a marine island situated in the southeast of Zhejiang, China, which may contribute to the difference from other areas.

The different concentrations of harmful metals in beans from various regions can be caused by several factors, including soil composition, climate conditions, and human actions. Human activities like industrial operations, farming methods, and mining endeavors have the potential to cause the accumulation of toxic metals in soil and water sources. Industrial operations, such as mining and manufacturing, release heavy metals like lead, mercury, and cadmium into the environment, contaminating soil and nearby water bodies. Farming methods that involve the use of chemical fertilizers and pesticides containing heavy metals can introduce these contaminants into the soil, leading to their accumulation over time. Improper waste disposal from various sources, including industries and households, can also contribute to the leaching of toxic metals into soil and water. Additionally, mining activities can release toxic metals into the environment, further exacerbating the contamination of soil and water sources. These human activities highlight the importance of implementing sustainable practices to minimize the accumulation of toxic metals in the environment and protect the health of ecosystems and human populations. To effectively address the potential health hazards, it is crucial to comprehend the underlying mechanisms behind their accumulation in different regions [35].

### 3.5. Health Risk Assessment

#### 3.5.1. Non-Carcinogenic Risks

The non-carcinogenic risk assessment for three groups (children, teens, and adults) was estimated by THQ (the ratio of EDI to the value of RfD). Its calculation was performed through a Monte Carlo simulation (MCS) with 100,000 iterations. Our results showed that THQs at the 50th percentile (P50) were all less than 1, which indicates there is no health risk from metal exposure with daily bean consumption (Table 4). The descending order is THQ-Cd > THQ-As > THQ-Hg/Pb > THQ-Cr for all groups. Furthermore, at the P95 level, THQs of Cd in children (0.88) and teens (0.82) were all greater than in adults (0.42). Actually, there has been growing concern regarding the presence of toxic elements, such as cadmium (Cd) and arsenic (As), in crops, particularly in Middle Eastern and Asian countries. For instance, a study conducted in Hunan, China, reported high median and mean THQs for Cd in rice, reaching 1.71 and 1.75, respectively [36]. Similarly, Ma et al. [23] investigated the THQ of Cd associated with rice consumption in Guangdong, China, revealing a mean THQ value of 1.43. These findings suggest a significant adverse health risk for local residents.

**Figure 3:**
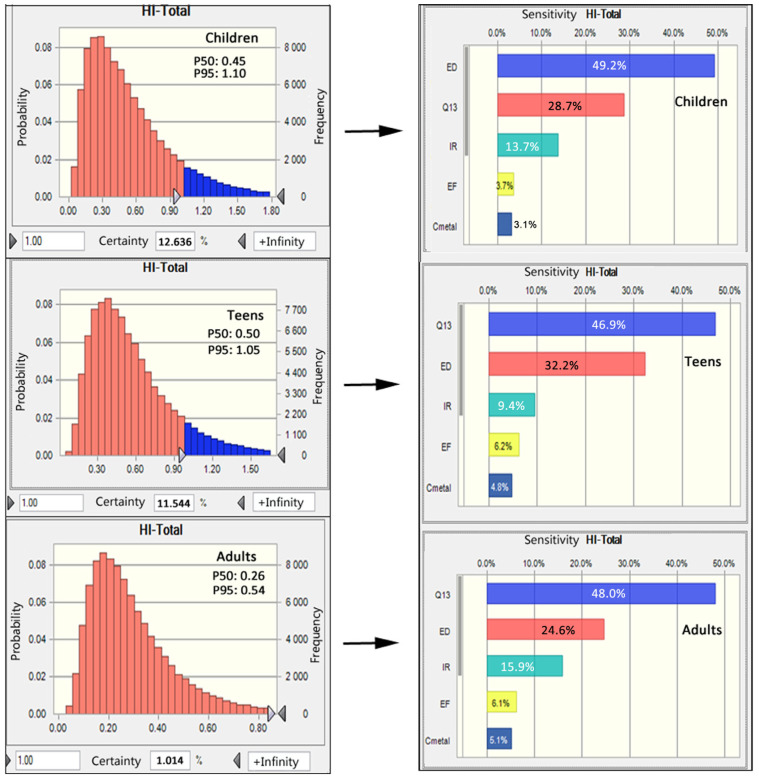
The cumulative distribution of the hazard indexes (HIs) and sensitivity analysis of toxic elements by Monte Carlo simulation.

For evaluating multiple elemental THQs, HI was adopted as the sum of the individual THQs. As shown in Figure 3, HIs at P50 were all less than 1 for all populations. But the HIs at P95 in children and teens were all more than 1. The certainty with HI greater than 1 for children was 12.64%, for teens 11.54%, and for adults 1.01%. It indicates it still has a potential health risk for bean consumption considering the toxicity of multiple elements. In addition, in order to figure out the main factor of health risk, a sensitivity analysis of non-carcinogenic risk was performed. The results revealed that two factors, the concentration of Cd in beans and ED (exposure duration), accounted for more than 70% of total HI values (Figure 3).

The methods of hazard index (HI) and target hazard quotient (THQ) have some limitations when used for health risk estimation related to heavy metal exposure through bean consumption. These limitations include simplified exposure assessment that may not accurately represent variations in consumption and metal levels, the lack of consideration for individual variability in factors like age, sex, and body weight, the focus on specific heavy metals while neglecting potential contaminants, uncertainties in toxicity values, and the failure to account for the long-term effects of chronic exposure. To improve accuracy, it is crucial to utilize more comprehensive and sophisticated risk assessment methods that address these limitations by incorporating individual variability, comprehensive metal analysis, accurate exposure assessment, and considering long-term cumulative effects.

#### 3.5.2. Carcinogenic Risks

**Figure 4:**
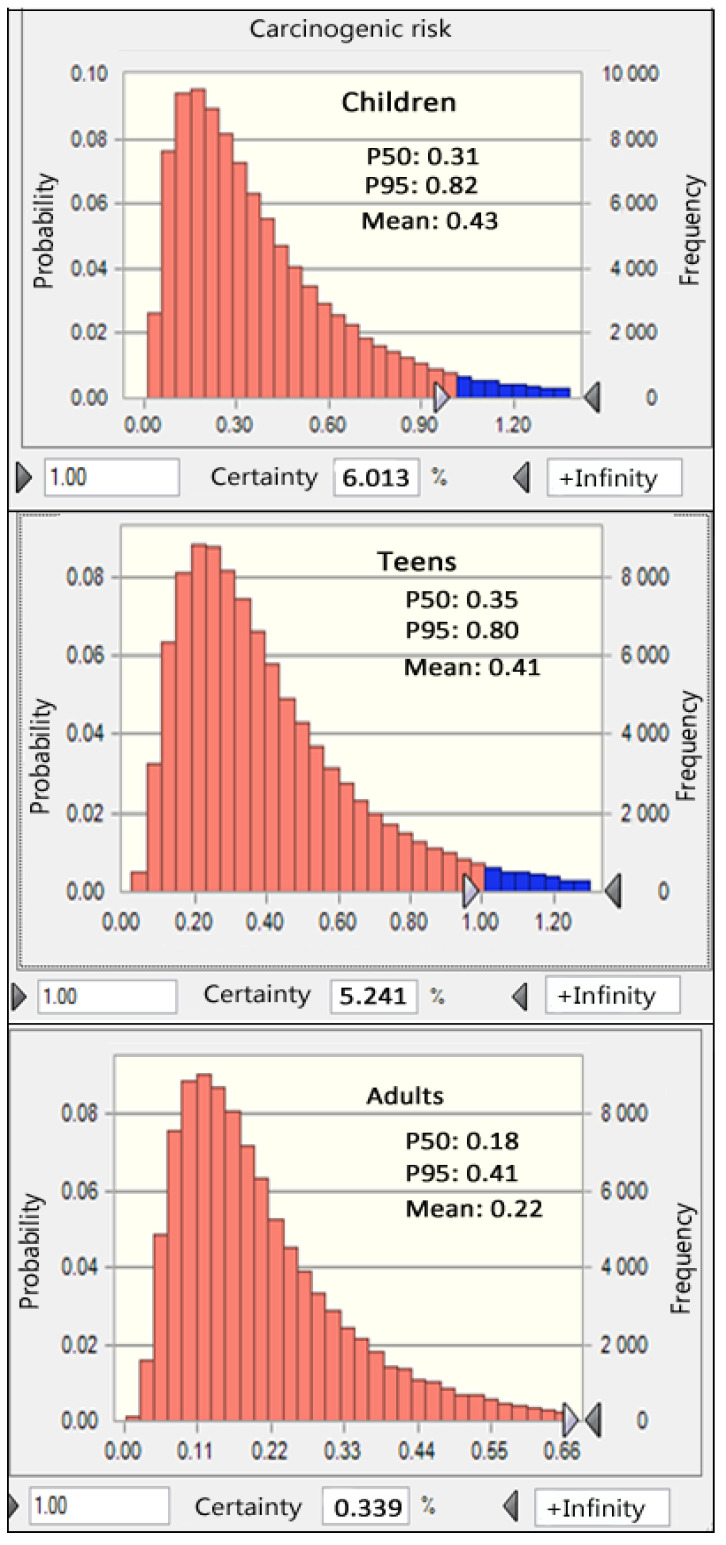
The cumulative distribution of carcinogenic risk (CR) of As for different populations obtained by Monte Carlo simulation.

Carcinogenic risk (CR) refers to the heightened probability of developing cancer as a consequence of exposure to carcinogens throughout an individual’s lifetime. The United States Environmental Protection Agency (USEPA) recommends an acceptable range for carcinogenic risk, ranging from 10^−6^ to 10^−4^. In our study, we specifically focused on evaluating the CR associated with arsenic, as the EPA’s Integrated Risk Information System (IRIS) database did not provide oral slope factors (SFs) for other elements. Our findings revealed that the average CRs for adults, teenagers, and children were 0.22 × 10^−4^, 0.41 × 10^−4^, and 0.43 × 10^−4^, respectively, as illustrated in Figure 4. All levels of CRs (P50, mean, and P95) did not exceed the threshold of 1 × 10^−4^. Furthermore, our analysis indicated that the probabilities of CR surpassing 1 × 10^−4^ were 0.34% for adults, 2.24% for teenagers, and 6.01% for children. These outcomes suggest there is no potential cancer risk from bean consumption. Another study was conducted by Yanez et al. [37], which reported a mean CR of 6.34 × 10^−4^ for arsenic detected in broad beans collected from Pastos Chicos, Argentina.

### 3.6. Uncertainty

The method of probabilistic health risk assessment is a valuable tool for evaluating the potential health risks associated with exposure to toxic elements. However, there are certain uncertainties and limitations that need to be considered when applying it to assess both non-carcinogenic and carcinogenic risks.

It relies on various input parameters such as exposure concentrations, toxicity values, and exposure duration. Estimating these parameters accurately can be challenging, as they often involve assumptions and extrapolations. The availability and quality of data can vary, leading to uncertainties in the risk assessment results. Additionally, PHRA assumes that exposure and toxicity follow probability distributions, but the selection and characterization of these distributions can introduce further uncertainty. The method typically focuses on individual risks and does not consider potential interactions or cumulative effects of multiple elements. The combined exposure to multiple chemicals can have synergistic or additive effects, which may not be adequately captured in a probabilistic framework. Furthermore, the current understanding of the toxicity and health effects of certain contaminants, especially for long-term exposure, may be limited. This can lead to uncertainties in the dose–response relationships and extrapolation of findings from animal studies to human health risks. Accordingly, uncertainties and limitations of the estimation model exist in estimating input parameters, characterizing probability distributions, and accounting for cumulative and interactive effects.

## 4. Conclusions

This study has revealed the distribution of As, Cd, Cr, Hg, and Pb in different beans from Zhejiang Province, southeast China. Strong positive correlations were found between the concentrations of Cd-Pb in kidney beans and mung beans, Cd-As in black beans, and Pb-As in red beans. Risk assessment with Monte Carlo simulation showed that the median THQs (P50) for five elements were all less than 1, which indicates a low health risk for local consumers. With respect to HIs (hazard indexes), the P95 levels in children and teens were all greater than 1, suggesting a potential health risk. Furthermore, the assessment of carcinogenic risks for As in beans showed no potential carcinogenic risk. However, it is still recommended that monitoring programs for Cd and Cr should be strengthened to ensure the safety of local consumers.

## Figures and Tables

**Table 1:** Analysis of elements in certified reference materials for rice.

Elements	Certified Values (mg/kg)	Tested (mg/kg)	LOQ (mg/kg)
As	0.035 ± 0.012	0.032 ± 0.015	0.0006
Cd	0.011	0.010 ± 0.005	0.0008
Cr	0.28 ± 0.04	0.26 ± 0.03	0.0006
Hg	0.0015	0.0012 ± 0.0005	0.0001
Pb	0.07 ± 0.02	0.06 ± 0.03	0.0008

**Table 2:** Parameters of Monte Carlo simulation for different populations in risk assessment.

Variance	Unit	Description	Distribution	Population		
				Children	Teens	Adults
*C*_metal_	mg/kg	Concentration of element	Log normal	analyzed based on different metals		
IR	g/day	Daily rice consumption	Normal	32.9	58.87	42.75
EF	day/year	Exposure frequency	Triangular	Min: 180; Mode: 345 Max: 365	Min: 180; Mode: 345 Max: 365	Min: 180; Mode: 345 Max: 365
ED	year	Exposure duration	Uniform	1–7	8–25	26–70
BW	kg	Body weight	Normal	16.68 ± 1.48	46.25 ± 1.18	57.03 ± 1.10
AT	days	Averaging time	Fixed	2190	5475	18,250
Rfd	mg/kg/day	Reference dose	Fixed	Varied based on different elements		

**Table 3:** The concentration of potential toxic elements in beans from Zhejiang province, southeast China.

Type	Elements	Concentration (mg/kg)					No. of >ML
		Median	Mean	Maxium	P95	SD	
Black bean (*n* = 307)	Cd	0.0336	0.0481	0.98	0.113	0.0481	4
	Cr	0.16	0.322	4.6	1.06	0.3220	11
	Hg	—	0.0019	0.037	0.00437	0.0019	—
	Pb	0.0045	0.0253	0.3	0.103	0.0253	3
	As	0.00987	0.0601	5.71	0.052	0.0601	—
Broad bean (*n* = 88)	Cd	0.005	0.0159	0.29	0.0448	0.0159	1
	Cr	0.1	0.18	1.05	0.67	0.1800	2
	Hg	—	0.00146	0.0059	0.00156	0.0015	—
	Pb	—	0.0188	0.135	0.0687	0.0188	0
	As	0.003	0.0197	0.29	0.083	0.0197	—
Mung bean (*n* = 43)	Cd	0.00409	0.0274	0.463	0.077	0.0274	2
	Cr	0.0721	0.101	0.379	0.241	0.1010	0
	Hg	—	0.000632	0.00165	0.00085	0.0006	—
	Pb	0.0139	0.0302	0.353	0.0677	0.0302	1
	As	—	0.0137	0.1	0.09	0.0137	—
Soybean (*n* = 104)	Cd	0.0226	0.0498	0.489	0.169	0.0498	4
	Cr	0.186	0.267	1.51	0.72	0.2670	3
	Hg	—	0.00213	0.0371	0.0088	0.0021	—
	Pb	—	0.0267	0.196	0.138	0.0267	0
	As	—	0.0133	0.17	0.05	0.0133	—
Red bean (*n* = 51)	Cd	0.00453	0.0301	0.648	0.177	0.0301	1
	Cr	0.126	0.164	0.965	0.436	0.1640	0
	Hg	—	0.0012	0.0143	0.00513	0.0012	—
	Pb	0.00861	0.0308	0.19	0.17	0.0308	0
	As	0.00613	0.0138	0.14	0.042	0.0138	—
Kidney bean (*n* = 12)	Cd	0.0165	0.0364	0.16	0.16	0.0364	0
	Cr	0.132	0.222	1.6	1.6	0.2220	1
	Hg	—	0.00258	0.0194	0.0194	0.0026	—
	Pb	0.0274	0.0498	0.183	0.183	0.0498	0
	As	—	0.0064	0.018	0.018	0.0064	—
Pea (*n* = 87)	Cd	0.019	0.0223	0.252	0.052	0.0223	1
	Cr	0.0669	0.152	1.18	0.66	0.1520	—
	Hg	—	0.00274	0.038	0.0079	0.0027	—
	Pb	—	0.0145	0.0757	0.05	0.0145	0
	As	—	0.0139	0.12	0.066	0.0139	—

Note: “No. of >ML” means the number of samples with an element level higher than the maximum limits; “—” means there is no related ML.

**Table 4:** The estimated target hazard quotients (THQs) of elements for different populations (Monte Carlo simulation).

Group	Type	As	Cd	Cr	Hg	Pb	Total (Sum)
Children	Mean	0.14	0.43	0	0.02	0.01	0.59
	P50	0.10	0.30	0	0.01	0.01	0.45
	P70	0.14	0.47	0	0.02	0.01	0.66
	P95	0.25	0.88	0	0.04	0.01	1.1
Teens	Mean	0.13	0.41		0.02	0.01	0.56
	P50	0.11	0.34	0	0.01	0.01	0.5
	P70	0.15	0.49	0	0.02	0.01	0.68
	P95	0.25	0.82	0	0.04	0.01	1.05
Adults	Mean	0.07	0.22	0	0.01	0	0.31
	P50	0.05	0.17	0	0.01	0	0.26
	P70	0.08	0.25	0	0.01	0	0.35
	P95	0.13	0.42	0	0.02	0.01	0.54

## Data Availability

The data used to support the findings of this study can be made available by the corresponding author upon request.

## Supplementary Materials

The following supporting information can be downloaded at: https://www.mdpi.com/article/10.3390/foods12173300/s1.

## References

[1] Pan X.-D., Han J.-L. (2023). Distribution of cadmium in fresh vegetables marketed in southeast China and its dietary exposure assessment. Foods.

[2] Ahmed S., Fatema Tuj Z., Mahdi M.M., Nurnabi M., Alam M.Z., Choudhury T.R. (2022). Health risk assessment for heavy metal accumulation in leafy vegetables grown on tannery effluent contaminated soil. Toxicol. Rep..

[3] Setia R., Dhaliwal S.S., Singh R., Kumar V., Taneja S., Kukal S.S., Pateriya B. (2021). Phytoavailability and human risk assessment of heavy metals in soils and food crops around Sutlej river, India. Chemosphere.

[4] Antoniadis V., Shaheen S.M., Boersch J., Frohne T., Du Laing G., Rinklebe J. (2017). Bioavailability and risk assessment of potentially toxic elements in garden edible vegetables and soils around a highly contaminated former mining area in germany. J. Environ. Manag..

[5] Conversa G., Miedico O., Chiaravalle A.E., Elia A. (2019). Heavy metal contents in green spears of asparagus (*Asparagus officinalis* L.) grown in Southern Italy: Variability among farms, genotypes and effect of soil mycorrhizal inoculation. Sci. Hortic..

[6] Miedico O., Iammarino M., Paglia G., Tarallo M., Mangiacotti M., Chiaravalle A.E. (2016). Environmental monitoring of the area surrounding oil wells in Val d’Agri (Italy): Element accumulation in bovine and ovine organs. Environ. Monit. Assess..

[7] Silva-Gigante M., Hinojosa-Reyes L., Rosas-Castor J.M., Quero-Jiménez P.C., Pino-Sandoval D.A., Guzmán-Mar J.L. (2023). Heavy metals and metalloids accumulation in common beans (*Phaseolus vulgaris* L.): A review. Chemosphere.

[8] Qureshi Y. (2021). Impact of heavy metals consumption on human health: A literature review. J. Pharm. Res. Int..

[9] Han J.-L., Pan X.-D., Chen Q., Huang B.-F. (2021). Health risk assessment of heavy metals in marine fish to the population in Zhejiang, China. Sci. Rep..

[10] Han J.L., Pan X.D., Chen Q. (2022). Distribution and safety assessment of heavy metals in fresh meat from Zhejiang, China. Sci. Rep..

[11] Chen Q., Pan X.-D., Huang B.-F., Han J.-L. (2018). Distribution of metals and metalloids in dried seaweeds and health risk to population in southeastern China. Sci. Rep..

[12] Huang Z., Pan X.-D., Wu P.-G., Han J.-L., Chen Q. (2014). Heavy metals in vegetables and the health risk to population in Zhejiang, China. Food Control.

[13] Pan X.-D., Han J.-L. (2023). Heavy metals accumulation in bivalve mollusks collected from coastal areas of southeast China. Mar. Pollut. Bull..

[14] Pan X.-D., Wu P.-G., Jiang X.-G. (2016). Levels and potential health risk of heavy metals in marketed vegetables in Zhejiang, China. Sci. Rep..

[15] WHO (1995). Reliable Evaluation of Low-Level Contaminants of Food. Workshop in the Frame of Gems/Food-Euro. http://www.Who.Int/foodsafety/publications/chem/en/lowlevel_may1995.Pdf.

[16] ZJFDA (2008). A Report on the Dietary Intake in Zhejiang Province, China. Hangzhou: Zjfda. http://www.Zjfda.Gov.Cn/news/detail/13556.Html.

[17] Wu B., Zhang Y., Zhang X.-X., Cheng S.-P. (2011). Health risk assessment of polycyclic aromatic hydrocarbons in the source water and drinking water of China: Quantitative analysis based on published monitoring data. Sci. Total Environ..

[18] Sanaei F., Amin M.M., Alavijeh Z.P., Esfahani R.A., Sadeghi M., Bandarrig N.S., Fatehizadeh A., Taheri E., Rezakazemi M. (2021). Health risk assessment of potentially toxic elements intake via food crops consumption: Monte carlo simulation-based probabilistic and heavy metal pollution index. Environ. Sci. Pollut. Res..

[19] Liu L., Han J., Xu X., Xu Z., Abeysinghe K.S., Atapattu A.J., De Silva P.M.C.S., Lu Q., Qiu G. (2020). Dietary exposure assessment of cadmium, arsenic, and lead in market rice from Sri Lanka. Environ. Sci. Pollut. Res..

[20] United States Environmental Protection Agency (2014). EPA Region 3 Riskbased Concentration Table. https://cfpub.Epa.Gov/ncea/iris/search/index.Cfm.

[21] FAO/WHO (2011). Evaluation of Certain Food Additives and Contaminants: Forty-First Report of the Joint Fao/Who Expert Committee on Food Additives. https://apps.Who.Int/iris/handle/10665/36981.

[22] Liang F., Li Y., Zhang G., Tan M., Lin J., Liu W., Li Y., Lu W. (2010). Total and speciated arsenic levels in rice from China. Food Addit. Contam..

[23] Ma L., Wang L., Tang J., Yang Z. (2017). Arsenic speciation and heavy metal distribution in polished rice grown in Guangdong province, southern China. Food Chem..

[24] Rajkumar H., Naik P.K., Singh G., Rishi M. (2022). Hydrogeochemical characterization, multi-exposure deterministic and probabilistic health hazard evaluation in groundwater in parts of northern India. Toxin Rev..

[25] Ades A., Cliffe S. (2002). Markov chain monte carlo estimation of a multiparameter decision model: Consistency of evidence and the accurate assessment of uncertainty. Med. Decis. Mak..

[26] Liu Z., Du Q., Guan Q., Luo H., Shan Y., Shao W. (2023). A monte carlo simulation-based health risk assessment of heavy metals in soils of an oasis agricultural region in northwest China. Sci. Total Environ..

[27] FAO/WHO (2000). Evaluation of certain food additives and contaminants. Geneva. World Health Organization, Joint Fao/Who Expert Committee on Food Additives.

[28] MHPRC (Ministry of Health of the People’s Republic of China) (2019). Maximum Levels of Contaminants in Foods (Gb2762–2019).

[29] Smith M., Mohammed F.K. (2020). Heavy metal intake and health risk implications from consumption of dried pulses in trinidad and tobago, W.I. Food. Addit. Contam. B.

[30] Hassan R.O., Othman H.O., Ali D.S., Abdullah F.O., Darwesh D.A. (2023). Assessment of the health risk posed by toxic metals in commonly consumed legume brands in Erbil, Iraq. J. Food Compos. Anal..

[31] Adepoju F.A., Adepoju A.F., Fadina O.O. (2018). Heavy metal levels in beans (*Vigna unguiculata*) in selected markets in Ibadan, Nigeria. J. Exp. Agric. Int..

[32] Islam M.S., Khanam M.S., Sarker N.I. (2018). Health risk assessment of metals transfer from soil to the edible part of some vegetables grown in Patuakhali province of Bangladesh. Arch. Agric. Environ. Sci..

[33] Karimi F., Shariatifar N., Rezaei M., Alikord M., Arabameri M. (2021). Quantitative measurement of toxic metals and assessment of health risk in agricultural products food from Markazi province of Iran. Int. J. Food Contam..

[34] Zhu Y., Yu H., Wang J., Fang W., Yuan J., Yang Z. (2007). Heavy metal accumulations of 24 asparagus bean cultivars grown in soil contaminated with cd alone and with multiple metals (cd, pb, and zn). J. Agric. Food. Chem..

[35] Yaashikaa P., Kumar P.S., Jeevanantham S., Saravanan R. (2022). A review on bioremediation approach for heavy metal detoxification and accumulation in plants. Environ. Pollut..

[36] Wang L., Ma L., Yang Z. (2018). Spatial variation and risk assessment of heavy metals in paddy rice from Hunan province, southern China. Int. J. Environ. Sci. Technol..

[37] Yañez L.M., Alfaro J.A., Avila Carreras N.M.E., Bovi Mitre G. (2019). Arsenic accumulation in lettuce (*Lactuca sativa* L.) and broad bean (*Vicia faba* L.) crops and its potential risk for human consumption. Heliyon.

