# Supplementary material for: Toxic Elements in Beans from Zhejiang, Southeast China: Distribution and Probabilistic Health Risk Assessment
Source: Foods. 2023 Sep 2;12(17):3300. doi: 10.3390/foods12173300 (PMC10486916; doi:10.3390/foods12173300)
Supplement: Supplementary file 1 [file foods-12-03300-s001.zip › foods-2578370-supplementary.pdf]

Supplemental tables

Table S1. The spearman correlation of multiple elements in all beans

	**Cd**	**Pb**	**Cr**	**Hg**	**As**
Cd	1				
Pb	0.399	1			
Cr	0.128	0.213	1		
Hg	0.068	0.292	0.103	1	
As	0.524	0.240	0.026	-0.014	1

Table S2. The spearman correlation of multiple elements in broad beans

	**Cd**	**Pb**	**Cr**	**Hg**	**As**
Cd	1				
Pb	0.241	1			
Cr	0.024	0.039	1		
Hg	0.090	0.133	0.227	1	
As	0.338	0.100	-0.138	0.094	1

Table S3. The spearman correlation of multiple elements in black beans

	**Cd**	**Pb**	**Cr**	**Hg**	**As**
Cd	1				
Pb	0.371	1			
Cr	0.110	0.240	1		
Hg	0.043	0.370	0.102	1	
As	0.720	0.349	0.017	-0.027	1

Table S4. The spearman correlation of multiple elements in red beans

	**Cd**	**Pb**	**Cr**	**Hg**	**As**
Cd	1				
Pb	0.332	1			
Cr	0.220	0.305	1		
Hg	0.182	0.338	0.685	1	
As	0.063	0.701	0.019	-0.070	1

Table S5. The spearman correlation of multiple elements in soybeans

	**Cd**	**Pb**	**Cr**	**Hg**	**As**
Cd	1				
Pb	0.308	1			
Cr	0.127	0.338	1		
Hg	0.032	0.444	0.051	1	
As	0.178	0.238	0.076	0.154	1

Table S6. The spearman correlation of multiple elements in mung beans

	**Cd**	**Pb**	**Cr**	**Hg**	**As**
Cd	1				
Pb	0.792	1			
Cr	0.310	0.512	1		
Hg	0.050	0.251	0.346	1	
As	0.114	0.138	-0.142	0.035	1

Table S7. The spearman correlation of multiple elements in peas

	**Cd**	**Pb**	**Cr**	**Hg**	**As**
Cd	1				
Pb	0.438	1			
Cr	0.078	0.183	1		
Hg	0.077	0.009	0.118	1	
As	0.007	0.251	0.084	-0.062	1

Table S8. The spearman correlation of multiple elements in kidney beans

	**Cd**	**Pb**	**Cr**	**Hg**	**As**
Cd	1				
Pb	0.934	1			
Cr	-0.062	-0.020	1		
Hg	0.746	0.592	0.027	1	
As	-0.175	-0.214	0.016	-0.120	1

Supplemental Table S9. The estimated target hazard quotients (THQs) of elements for different population (Monte Carlo simulation).

		As	Cd	Cr	Hg	Pb	Total (sum)
Children	Mean						
	P50	0.10	0.30	0	0.01	0.01	0.46
	P70	0.14	0.47	0	0.02	0.01	0.66
	P95	0.25	0.88	0	0.04	0.01	1.1
Teens	Mean						
	P50	0.11	0.34	0	0.01	0.01	0.5
	P70	0.15	0.49	0	0.02	0.01	0.68
	P95	0.25	0.82	0	0.04	0.01	1.05
Adults	Mean						
	P50	0.05	0.17	0	0.01	0	0.26
	P70	0.08	0.25	0	0.01	0	0.35
	P95	0.13	0.42	0	0.02	0.01	0.54
